# Digital communication and virtual reality for extending the behavioural treatment of obesity – the patients’ perspective: results of an online survey in Germany

**DOI:** 10.1186/s12911-023-02197-1

**Published:** 2023-05-24

**Authors:** Claudia Luck-Sikorski, Regine Hochrein, Nina Döllinger, Carolin Wienrich, Kathrin Gemesi, Sophie Holzmann, Christina Holzapfel, Natascha-Alexandra Weinberger

**Affiliations:** 1 Research Group “Chronic Diseases and Psychological Health” (COPE), SRH University of Applied Health Sciences, Neue Str. 28-30, 07548 Gera, Germany; 2 Human-Technology-Systems, University of Würzburg, Würzburg, Germany; 3 School of Medicine, Institute for Nutritional Medicine, Technical University of Munich, Munich, Germany

**Keywords:** Obesity, Virtual reality, Attitudes, Treatment

## Abstract

**Background:**

CBT has been found effective for the treatment of EDs and obesity. However not all patients achieve clinically significant weight loss and weight regain is common. In this context, technology-based interventions can be used to enhance traditional CBT but are not yet widespread. This survey therefore explores the status quo of pathways of communication between patients and therapists, the use of digital applications for therapy as well as attitudes towards VR from the perspective of patients with obesity in Germany.

**Methods:**

This cross-sectional online survey was conducted in October 2020. Participants were recruited digitally through social media, obesity associations and self-help groups. The standardized questionnaire included items concerning current treatment, paths of communication with their therapists, and attitudes toward VR. The descriptive analyses were performed with Stata.

**Results:**

The 152 participants were mostly female (90%), had a mean age of 46.5 years (*SD* = 9.2) and an average BMI of 43.0 kg/m² (*SD* = 8.4). Face-to-face communication with their therapist was considered of high importance in current treatment (*M* = 4.30; *SD* = 0.86) and messenger apps were the most frequently used digital application for communication. Participants were mostly neutral regarding the inclusion of VR methods in obesity treatment (*M* = 3.27; *SD* = 1.19). Only one participant had already used VR glasses as part of treatment. Participants considered VR suitable for exercises promoting body image change (*M* = 3.40; *SD* = 1.02).

**Discussion:**

Technological approaches in obesity therapy are not widespread. Face-to-face communication remains the most important setting for treatment. Participants had low familiarity with VR but a neutral to positive attitude toward the technology. Further studies are needed to provide a clearer picture of potential treatment barriers or educational needs and to facilitate the transfer of developed VR systems into clinical practice.

**Supplementary Information:**

The online version contains supplementary material available at 10.1186/s12911-023-02197-1.

## Abstract (plain language summary)

CBT is the treatment of choice for a variety of psychological disorders, among them eating disorders and obesity. However not all patients benefit equally and weight-regain is frequent. Technology-based approaches like virtual reality can enhance traditional CBT, but they are not yet used very often in clinical practice. This study asked patients with obesity which digital methods of communication and treatment were part of their obesity therapy and whether they would welcome VR approaches in their therapy. 152 patients participated and reported a face-to-face communication with their therapist to be of high importance. Messenger apps (e.g. WhatsApp) were the most commonly used digital methods of communication. VR applications or wearables do not play a role in the respondents’ therapy. Participants were mostly neutral toward VR technology but expressed positive expectations toward VR as part of body image therapy.

**Supplementary Information:**

The online version contains supplementary material available at 10.1186/s12911-023-02197-1.

## Background

Obesity is defined as a Body Mass Index (BMI) of greater than 30 kg/m². The chronic condition poses one of the major health care challenges throughout the world, with prevalence rates reaching up to 40% of the adult population in some countries [1], with the COVID-19 pandemic further contributing to weight gain, unhealthy eating behaviour and declines in metabolic health [2–4].

As individuals with obesity are faced with a number of adverse effects, such as cardiac disease, a higher risk for cancer and psychological disorders [5] and overall increased mortality [6], treatment and intervention programs are essential. Evidence-based guidelines by national and international obesity societies (e.g., German Obesity Association, DAG; European obesity community, EASO) outline lifestyle-based interventions for the management of overweight and obesity in adults [7]. Next to the increase of physical activity and the reduction of energy intake, the use of behavioural weight loss approaches like Cognitive Behavioural Therapy (CBT) are recommended not least due to the high prevalence of comorbidities like depression and binge eating disorder (BED; [8]; [9]. CBT has been established as the method of choice for the treatment of various psychological disorders like depression [10] anxiety [11] or eating disorders (ED; [12, 13] and particularly within the context of ED and obesity therapy typically includes elements like stimulus control, goal setting, cognitive restructuring, and reinforcement [14, 15].

While CBT has been shown to be efficacious for the treatment of EDs and obesity [16, 17], it does not necessarily result in weight loss in all patients and weight-regain is frequent especially over the long term [18–20].

Consequently, a growing body of research over the years has examined further development and/or adaptation of traditional CBT programs such as “enhanced” CBT approaches (e.g. CBT-Ef/CBT-Eb; [21]), mindfulness-based interventions like for example acceptance and commitment therapy (ACT; [22, 23] as well as the use of advanced technologies such as virtual reality (VR; [24].

VR technology is able to simulate a variety of situations and settings close to the real-world under controlled conditions [25]. In addition, VR permits a higher degree of immersion compared to for example imagery exposure and can facilitate patients’ emotional involvement [26]. The development and evaluation of immersive technologies could help address the frequently observed gap between behavioural intention and actual behaviour in current obesity management [27]. The Behavioural Framework of Immersive Technologies (BehaveFIT) outlines how VR could help overcome psychological barriers [28] and previous studies have found VR to be effective in terms of changing behaviour and lifestyle [29, 30]. Classic CBT approaches have seen an introduction of VR to enhance treatment effects across certain indications, such as anxiety disorders [31]. These have shown to be at least equally effective compared to exposure in person [32]. In the treatment of obesity, VR has been used to address food and eating behaviour as well as body image perceptions by immersing patients in realistic avatars in virtual scenarios of every-day situations like e.g. grocery shopping [33–35]. In a study by Manzoni and colleagues, women with obesity completed the treatment either in the standard care arm, standard care plus CBT or standard care plus CBT and VR-enhancement [36]. At the one-year follow up, only women in the VR-enhanced CBT group further improved their weight loss compared to the other two groups. Riva and colleagues found a short-term VR therapy to be more effective than traditional CBT in reducing body dissatisfaction [37]. While further studies are needed, VR interventions have also shown promise in the treatment of obesity risk factors such as smoking, alcohol consumption, nutrition and physical activity [38, 39] and could even be utilized in the care of bariatric patients undergoing surgery [40].

In Europe up to date, VR in the treatment of EDs and obesity has seen extensive research particularly in Italy and Spain [26]. It is unclear to what extent VR technology might be a part of lifestyle-based treatments in Germany and whether VR methods would be welcomed by therapists and patients. A recent study with nutrition experts shows that very few have ever used VR technology in their daily treatment environment and that attitudes towards the importance of VR technology were neutral [41]. A body of evidence around health care professionals’ attitudes towards teletherapy and digitalization exists, but few studies focus on the patients that would be receiving treatment with new technologies. Patient-centred approaches, however, are necessary to ensure acceptance of new methods in treatment. For instance, smartphone apps showed to be a facilitator of health literacy [42]. In Britain, a study in primary care patients documented that knowledge of digital resources and technology were low, but patients were generally positive toward that option [43]. Without a generally positive mindset of patients toward new technology in treatment, barriers for the use of VR are high. Therefore, investigating which technologies patients already know and use in therapy and what their general attitude toward new methods is, could help to introduce new approaches more effectively.

The current study is part of the German ViTraS project (Virtual Reality Therapy by Stimulation of Modulated Body Image). The project develops and investigates therapy methods for obesity based on controlled modulation of body perception and behaviour patterns with the help of current VR technologies and follows the approach of enhancing standard behavioural treatment by combining CBT and VR methods [44]. In this context, the survey explores the status quo of pathways of communication between patients and therapists, the use of digital applications for therapy as well as attitudes toward VR from the perspective of patients with obesity.

## Methods

### Design

This cross-sectional survey was performed in October 2020 throughout Germany. The Ethical Committees of the Friedrich-Schiller-University Jena and the School of Medicine at the Technical University of Munich approved this open online survey (ethical vote: 410/20S, 2020-1885-Bef). Written informed consent was given by all participants when starting the online survey. All methods were carried out in accordance with relevant guidelines and regulations. The survey invitation included a link that guided participants to the online survey on the platform SoSci Survey [45]. All participants gave informed consent before participation and had to confirm the data privacy statement. No incentives were offered to the participants.

The recruitment was conducted mainly through expert associations (e.g. the German obesity association), social media (e.g. Facebook), and self-help groups. Included participants had to be over the age of 18, to have good German language skills and a Body Mass Index (BMI) of ≥ 30 kg/m². Eligibility was checked in the beginning of the survey (BMI calculated via self-reported height and weight). Due to the electronic delivery of the survey invitation, the exact number of invitations and the response rate is unknown.

### Questionnaire

The 56-item questionnaire was developed by a multidisciplinary team of computer scientists, psychologists, and nutritionists (for an English language version of the patient version of the questionnaire see Additional file 1). The survey started with an introduction and information about data privacy and protection. All technical terms including VR terminology in the questionnaire were explained in plain language and in addition illustrated with graphics where appropriate (e.g. picture of VR glasses; picture of a 3D-avatar in an VR environment). Moreover, patients pre-tested the first draft of the questionnaire to report any problems of comprehension.

After pretesting, the questionnaire for the current study included (closed, open, single, or multiple choice) questions referring to their current treatment (type, setting and location of therapy), the relevance of different paths of communication with their therapists and other patients, as well as VR-related questions (their general attitude towards VR in therapy, their preferred setting for VR therapy, their preferred role of the therapist in VR environments, their general attitude towards the use of VR glasses, their general attitude towards the use of VR as part of body image therapy, and what they personally consider to be advantages and disadvantages of VR as part of therapy). Moreover, the frequency of and their satisfaction with behavioural treatment techniques, sociodemographic data (age, gender, and education), obesity-related information (weight, height, plans to undergo bariatric surgery and comorbidities), and general feedback to the survey was recorded. Neutral answer options like “occasional,” “other,” or “neutral” were provided if not indicated otherwise. Items not presented in the current paper focused on nutritional questions and technical aspects for the design and development of the VR environment within the ViTraS project.

### Statistical analyses

All statistical analyses were performed with Stata SE 14 [46]. Integrity and plausibility of all data was checked. Since participants were able to quit the survey at any time, sample size differs between questionnaire items. Sociodemographic data was compared across BMI categories using Chi² tests. Descriptive statistics (M, SD, %) are given for all questions. No gender specific analyses were performed (90% women). P-values < 0.05 were considered as statistically significant.

## Results

The final sample consisted of 152 participants, with 136 (90%) being female. On average, participants reported a BMI of 43.0 kg/m² (*SD* = 8.4). Table 1 summarizes participants’ characteristics across the three different BMI classes. Differences across the groups were observed regarding age and plans to undergo metabolic surgery.

**Table 1:** *Descriptive statistics (means and standard deviations) of gender, age, education, present comorbidities and plans to undergo metabolic surgery across BMI category*

	Total (*N* = 152)	Class 1 (*n* = 30)	Class 2 (*n* = 29)	Class 3 (*n* = 93)	difference
	*M (SD)*	*M (SD)*	*M (SD)*	*M (SD)*	*p*
gender^a^	1.90 (0.30)	1.90 (0.31)	1.83 (0.38)	1.92 (0.27)	0.319
% men	9.9	10.0	17.2	7.6	-
% women	90.1	90.0	82.8	92.4	-
age	46.50 (9.19)	50.20 (9.73)	47.10 (9.12)	45.12 (8.77)	0.032*
education^b^	3.51 (1.14)	3.47 (0.94)	3.55 (1.21)	3.52 (1.19)	0.570
comorbidities^c^	2.76 (1.62)	2.45 (1.57)	2.64 (1.52)	2.90 (1.67)	-
% binge eating syndrome	18.9	17.2	14.3	20.9	0.714
% night eating syndrome	7.4	6.9	10.7	6.6	0.762
% type 2 diabetes mellitus	20.3	17.2	21.4	20.9	0.901
% hypertension	38.8	37.9	25.0	45.1	0.161
% anxiety disorder	14.2	13.8	21.4	12.1	0.463
% depression	36.5	34.5	35.7	37.4	0.957
Metabolic surgery^d^	1.24 (0.72)	2.00 (0.00)	1.39 (0.85)	1.03 (0.64)	0.001***
% not planned or undergone	9.9	-	13.8	11.8	-
% planned	25.7	-	10.3	38.7	-
% undergone	24.3	43.3	37.9	14.0	-

*Note*. BMI classes according to WHO: obesity class 1 = 30.0–34.9 kg/m²; obesity class 2 = 35.0–39.9 kg/m²; obesity class 3 = ≥ 40 kg/m². Comparisons based on Chi-Squared tests

^a^1=male, 2 = female. ^b^range 1 = eighth grade or less to 5 = college degree. ^C^number of comorbidities reported. ^d^range 0 = not planned or undergone, 1 = planned, 2 = undergone

*p < .05. ***p < .001

At the time of the survey about 38.7% of participants were not in treatment for their obesity. Participants in treatment were most frequently enrolled in nutritional therapy (48%), psychotherapy (43.3%) and/or physical therapy (26%). An individual therapy setting was reported by 38.7% of participants, while 23.1% attended group therapy. The most frequently reported therapy locations were psychotherapy practices (55.4%), practices for nutrition therapy (44.6%) and outpatient facilities (31.5%), with inpatient facilities (4.3%), rehabilitation clinics and counselling centres (7.6% each) only playing a tangential role.

Participants rated face-to-face communication with their therapist as well as with other patients to be of high importance in their treatment. Table 2 illustrates the descriptive statistics of all ways of communication.

**Table 2:** *Importance of different ways of communication in the context of participants’ obesity treatment*

	Communication with therapist		Communication with other patients	
	*M*	*SD*	*M*	*SD*
face-to-face	4.30	0.86	3.59	1.20
via telephone	3.49	0.99	2.92	1.13
via E-Mail	3.42	1.20	3.01	1.15
via video	3.03	1.27	2.72	1.19

*Note*. n = 141. Higher scores indicate higher importance: 1 = not important to 5 = very important

**Fig. 1:**
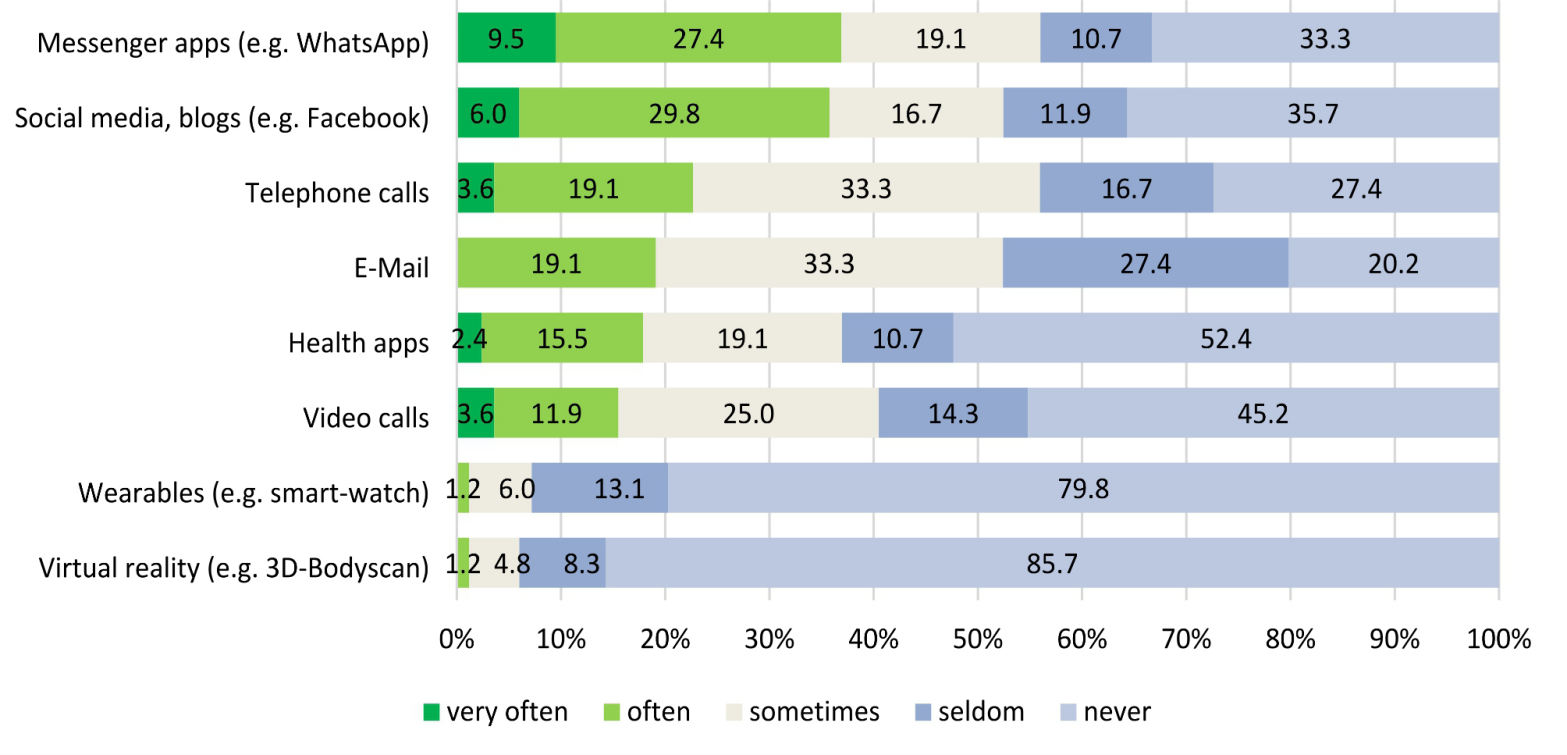
Frequency of use of digital and/or virtual reality applications in participants’ obesity treatment (*n* = 85)

Further, most frequently used digital and/or virtual reality applications were found to be messenger apps like e.g. WhatsApp, social media apps like e.g. Facebook, and telephone calls. Figure 1 illustrates the reported frequency of use for all possible answer categories.

**Fig. 2:**
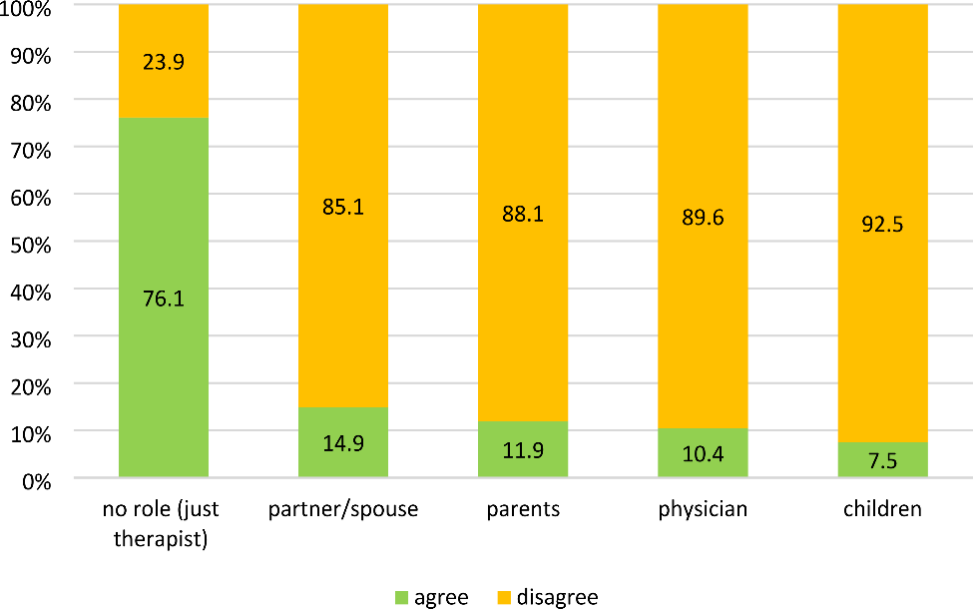
Responses to the question *“In the context of virtual realities, it is possible for your therapist to “slip into” a virtual body. The therapist can then talk to you in real time (live), and you can see his/her facial expressions and gestures. Your therapist could also take on a different role for the counselling situation. What roles can you imagine for your therapist?”* (*n* = 67)

Participants were mostly neutral regarding the inclusion of VR methods in obesity treatment (*M* = 3.27; *SD* = 1.19). Moreover, an individual therapy setting for the use of VR was considered suitable (*M* = 4.00; *SD* = 1.61) while participants were more neutral with regards to the use of VR for group therapy (*M* = 2.62; *SD* = 0.87). Moreover, the majority of participants did not want for their therapist to play a different role during VR therapy sessions (see Fig. 2 for details).

Furthermore, only one out of 136 participants who responded to the question reported that VR glasses had been used as part of their obesity treatment. As Table 3 also illustrates, participants’ attitudes toward the use of VR glasses as part of their treatment were neutral.

**Table 3:** *Attitudes toward the use of VR glasses as part of participants’ obesity treatment*

	*M*	*SD*
I have already used VR glasses as part of my current therapy.^a^	1.99	0.09
It would be easy for me to use VR glasses.^b^	3.24	1.34
I would like to use VR glasses as part of the treatment.^b^	3.41	1.43

*Note*. n = 136

^a^1 = yes, 2 = no. ^b^higher scores indicate higher agreement: 1 = not applicable to 5 = applicable

Also, participants considered the use of VR suitable to support exercises intended to change their body image perception (*M* = 3.40; *SD* = 1.02). Further, Table 4 illustrates that overall, the frequency of behavioural (body image) treatment techniques in participants’ current treatment was low, while satisfaction with the treatment techniques was judged as low or neutral.

**Table 4:** *Frequency of and satisfaction with behavioural treatment techniques of participants’ current treatment*

	Frequency^a^		Satisfaction^b^	
	*M*	*SD*	*M*	*SD*
Imagination exercises (e.g. going out to eat)	2.09	1.14	2.82	1.31
Exercises in everyday situations	2.03	1.15	2.75	1.30
Exercises with/in front of a mirror	1.65	0.94	2.34	1.19
Touching one’s own body	1.63	0.90	2.36	1.12
Drawing of own body	1.37	0.75	2.26	1.14
Exercises with video recordings of own body	1.33	0.66	2.33	1.15
Exercises with modelling material (e.g. clay, plasticine)	1.20	0.48	2.31	1.10

*Note*. n = 86

^a^Higher scores indicate higher frequency of use: 1 = never to 5 = very often. ^b^Higher scores indicate higher satisfaction: 1 = not satisfied to 5 = very satisfied

Finally, a majority of participants made use of the open response options to share what they personally consider to be advantages and disadvantages of VR as a part of obesity therapy. Table 5 describes the most frequently commented responses.

**Table 5:** *Advantages and disadvantages of VR as part of obesity therapy from the patients’ perspective*

Advantages^a^		Disadvantages^b^	
*response*	*number of mentions*	*response*	*number of mentions*
promotes self-perception/body esteem	23	lack of direct human contact	37
high practical relevance and/or realism	20	general aversion to/unawareness of technology	17
helps with visualisation	20	feelings of unreality	16
high flexibility/individuality	15	lack of sensory impressions and/or emotions	11
promotes motivation	9	might evoke negative feelings^c^	10
provides safe space	8	technical difficulties	7
news value	5	difficulty getting used to VR	6
personal interest in VR	2	fear of high treatment costs	5
no advantages	38	no disadvantages	36
do not know	14	do not know	13

^a^Number of responses = 108. ^b^ Number of responses = 110. ^C^for example anxiety/panic attacks, body dissatisfaction

## Discussion

This survey reports the use of digital methods in therapy and attitudes toward VR in a predominantly female sample of individuals with obesity. While a high prevalence of psychiatric comorbidities was observed, 38.7% of participants were currently not in treatment. Those in treatment were most frequently enrolled in nutritional therapy or psychotherapy. Participants only rated face-to-face communication with their therapist to be of high importance in their treatment. The most frequently used digital applications were found to be messenger apps like WhatsApp, social media apps like Facebook, and telephone calls. Only one participant reported that VR glasses had already been used as part of their obesity treatment. Further, participants were mostly neutral regarding the inclusion of VR methods in obesity treatment but considered the use of VR suitable to support exercises intended to change their body image perception. The majority of participants preferred their therapist to keep the role as therapist in the VR environment (e.g. as part of a role-playing exercise). Moreover, an individual therapy setting for the use of VR was considered suitable while participants were more neutral with regards to the use of VR for group therapy. While a high number of responses fell into the “I do not know” / “No (dis-)advantages” categories, participants most frequently considered the lack of direct human interaction, general aversion to or unawareness of technology, and feelings of unreality to be disadvantages of VR as a part of obesity therapy. Finally, promotion of self-perception and/or body esteem, a high practical relevance and/or realism, and the help with visualisation were frequently considered to be advantages.

Regarding frequency of use of digital technology as part of obesity treatment, similar results have been reported in previous studies: Horne and colleagues for example state that fewer than 20% of individuals using aids to assist weight management in England reported using digital technologies such as wearable trackers, mobile phone applications or websites [34]. In line with this, Solbrig and colleagues discuss willingness to engage with such technology in people trying to lose weight and suggest that motivational digital technologies in particular are required to support them [47]. Moreover, the importance participants’ ascribed to face-to-face interactions with their therapist and their hesitance to see their therapist potentially take on a different role in VR environments does not surprise considering the role of the patient-therapist-relationship particularly in psychological therapy (e.g. [48]. In CBT, this therapeutic alliance has been found to be a prerequisite for the adherence and competence of implementing therapeutic techniques and even a predictor of treatment failure [49].

Further, the rare use of VR as part of obesity treatment and neutral stance of the current participants with regard to its relevance are similar to a recent study in nutritional therapists who rated the suitability and importance of VR in the treatment of obesity as neutral [41]. The results are also in line with the general state of research and consequently implementation of the technology into standard care: Different reviews of VR in the context of obesity have pointed out the heterogeneity and limited number of studies [34, 38, 50]. As such, VR is considered an emerging technology [51]. Its low prevalence in treatment is not surprising considering for example the high costs of VR equipment and its maintenance, as well as VR software development [52], and open questions regarding the extent of acceptance in both, patients and therapists [53]. A survey with psychotherapists who were not using VR in their therapy at the time of the study found that they were not familiar with the benefits and applications of VR in treatment and did not voice much interest in VR in clinical practice because of possible costs and the need for extra training [54]. In contrast, a more recent study by Lindner and colleagues concludes that attitudes among practicing CBT therapists toward VR do not constitute a major barrier to implementing VR technology into clinical practice anymore [55]. In line with these findings, acceptance of VR technology tends to be high in patients (e.g. [53, 56, 57], although they also have expressed data security concerns in the past [58].

While behavioural body image techniques were rarely used in their current treatment, participants in our study considered VR to be of potential use in supporting exercises intended to change their body image perception. Impaired body image has been documented for women with obesity in particular [59] and VR approaches are considered to be an alternative to e.g. guided imagery and in vivo exposure in this context [26]. VR interventions have been found to be effective in improving not just body image concerns, but also health self-efficacy, and motivation to change [51], [60], [61]. In addition, VR can provide a safe environment for users to confront feared situations and thus reduce their avoidance [26], which is especially relevant in patients with obesity who often experience shame [62] and stigmatisation in their daily lives and health care settings [63].

### Strengths and limitations

This study is the first to shed light on the status quo of means of communication and digitalization as well as participants’ attitudes toward VR approaches as part of their treatment. It gathered data from a German sample of people with obesity that included all three obesity classes as defined by the WHO [64].

While it is known that psychiatric comorbidities are more prevalent in people with obesity [65], the amount of respondents that reported suffering from depression or anxiety in the current sample is startling. This finding leads to two main implications: For one, the current results should not be generalised to the total population of individuals with obesity: It seems likely that individuals with obesity suffering considerable physical or psychological strain felt more drawn to the survey and its topic than participants with obesity who consider themselves healthy or do not seek treatment. Secondly, the finding further emphasizes the relevance and importance of cognitive behavioural therapy in the treatment of patients with obesity. Furthermore, the very low participation of men in the current study mirrors previous findings [34, 51, 66]. While one common assumption for the often-observed gender difference in recruitment is the higher societal pressure for women to conform to thin body ideals and the consequently higher psychological strain leading them to seek treatment more frequently (34), further causes remain unclear and should be addressed in future studies to avoid a potential healthcare gap.

Moreover, due to time constraints with regards to the survey and its exploratory nature, only a limited number of questions spanning the three fields of expertise (nutrition, VR technology, psychology) could be included and no standardized questionnaires were used. As such, the final questionnaire did not include items to investigate the disadvantages of VR applications like cybersickness or digital literacy. While it is important to consider in the design of virtual environments, previous studies on the topic conclude that cases of cybersickness associated with exposure to VR environments are rare [26]. Regarding digital literacy, existing research reports socioeconomic status (SES) and age differences in equality of access and competence [67, 68]. Due to a higher vulnerability to obesity in low-SES subgroups of the population in developed countries [69], future studies investigating the associations between SES and digital literacy in the context of VR therapy could provide a clearer picture of potential treatment barriers or educational needs.

Similarly, while precautions were taken to ensure a high level of comprehension regarding all technical terminology (plain language explanations as well as use of graphics; pre-test of the questionnaire with patients with obesity), the high proportion of “neutral” answers could imply that participants might nevertheless have been out of their depth when faced with the topic of VR. Future studies about the development or the evaluation of VR treatment methods might need different or more direct (e.g. qualitative studies; demonstrations of the VR exercises of interest) approaches to gauge interest in or acceptance of VR therapy methods and to ensure that a broad variety of patients with obesity are included.

## Conclusion

In this first survey of the status quo and general attitudes toward technological approaches in obesity therapy in Germany, participants with obesity had a neutral to positive attitude to VR but were not familiar with the technology. Face-to-face communication remains the most important setting for treatment and VR approaches are not part of treatment in the vast majority of cases. Particularly for the highly stigmatized group of individuals with obesity, VR therapy could offer a safe environment to confront stressful situations that are contributing to weight gain and research of VR as part of obesity treatment has shown promising results regarding health behaviour change [29, 70] and self-monitoring of diet and physical activity, improving body image and supporting weight loss in the treatment of overweight and obesity [51]. Further studies are needed however to provide a clearer picture of potential treatment barriers or educational needs in different subpopulations of persons with obesity and to facilitate the transfer of developed VR systems into clinical practice.

## Electronic supplementary material

Below is the link to the electronic supplementary material.

Supplementary Material 1

## Data Availability

All data generated or analysed during this study are included in this article. Further enquiries can be directed to the corresponding author.
