# Supplementary material for: Digital communication and virtual reality for extending the behavioural treatment of obesity – the patients’ perspective: results of an online survey in Germany
Source: BMC Med Inform Decis Mak. 2023 May 24;23:100. doi: 10.1186/s12911-023-02197-1 (PMC10206569; doi:10.1186/s12911-023-02197-1)
Supplement: Supplementary file 1 — Supplementary Material 1 [file 12911_2023_2197_MOESM1_ESM.docx]

**ViTraS: Virtual Reality Therapy by Stimulation of Modulated Body Perception**

*Multi-methodological study to investigate the behavioural therapeutic use of virtual reality (VR) - Applications in the psychotherapeutic and nutritional therapy treatment of persons with overweight and obesity - a bilateral needs analysis (experts/patients)*

**Survey on the use of VR (virtual reality) in the treatment of body image and body perception in people with overweight and obesity** **- Needs assessment questionnaire for patients:**

**Height and Weight**

**What is your height in centimetres (cm)?**

**What is your current** **weight** **in kilograms (kg)?**

**Your BMI is: __ kg/m^2^ (calculated via formula; end of survey if BMI < 30)**

**My highest weight so far has been:** _________kilograms at the age of _____ years.

**How long have you been overweight or obese?**  *Please tick only* ***one*** *answer!*

|  | Childhood (under 14 years) |
| --- | --- |

|  | Youth (14 to 21 years) |
| --- | --- |
|  | Adulthood (from the age of 21) |

**Treatment**

**Are you currently** **undergoing one of the following treatments because of your weight?**

|  | **Yes** | **No** |
| --- | --- | --- |
| Nutritional therapy |  |  |
| Psychotherapy |  |  |
| Physical therapy |  |  |

**Where does the treatment take place?**

| Clinic/hospital (outpatient = no overnight stay in the facility) |  |
| --- | --- |
| Clinic/hospital (inpatient = with overnight stay in the facility) |  |
| Rehabilitation centre/clinic |  |
| Psychotherapeutic practice |  |
| Practice for nutritional counselling, nutrition medicine or -therapy |  |
| counselling centre |  |
| Other: FREE TEXT |  |

**What applies to you?**

*I am currently in...*

|  | Individual counselling/therapy: I am alone with my therapist. |
| --- | --- |
|  | Group counselling/therapy: I am in a group with several patients and my therapist. |

**What applies to you?**

I am currently planning a surgical procedure (= metabolic surgery) to treat my obesity.

I already had a surgical procedure (= metabolic surgery) to treat my obesity.

No answer applies.

**Which of the following illnesses are you currently affected by?**  ***Several*** *crosses possible!*

| \|  \| Anorexia nervosa \| \| --- \| --- \| \|  \| Bulimia nervosa \| \|  \| Binge eating syndrome \| \|  \| Nocturnal Binge Eating (Night Eating Syndrome) \| \|  \| Body schema disorder (distorted perception of one's own body) \| \|  \| Thyroid disease \| \|  \| Malnutrition \| \|  \| Diabetes mellitus (Type 2 diabetes mellitus) \| \|  \| Lipometabolic disorder \| \|  \| Hypertension \| \|  \| Cancer \| \|  \| Anxiety \| \|  \| Depression (affective disorders) \| \|  \| Disorders caused by alcohol and drug use \| \|  \| Diseases of the gastrointestinal tract \| \|  \| Allergies/food intolerances \| \|  \| none of the above-mentioned illnesses \| \|  \| Other: FREE TEXT \| |  |
| --- | --- | --- | --- | --- | --- | --- | --- | --- | --- | --- | --- | --- | --- | --- | --- | --- | --- | --- | --- | --- | --- | --- | --- | --- | --- | --- | --- | --- | --- | --- | --- | --- | --- | --- | --- | --- | --- |

| **Body image** |
| --- |

**Work on body image can be part of the therapy of overweight/obesity. Body image is the perception and attitude of a person to their own body and appearance. We now want to find out what role body image plays in the treatment of your weight.**

**How often are the following exercises used in the treatment of your weight? (Please pick only one frequency per answer.) Answer options in survey: never, rarely, occasionally, often, very often**

- Touching one's own body
- Drawing one's own body
- Exercises with modelling clay (e.g. clay, modelling clay)
- Exercises with/in front of a mirror
- Exercises with video recordings of one's own body
- Exercises in everyday situations
- Exercises in which you imagine something (e.g. eating out)
- Other FREE TEXT

No exercises are done.

**How satisfied are you with the exercises you did as part of your treatment? (Please pick only one answer per exercise!) Answer options in survey: not satisfied, rather not satisfied, neutral, satisfied, very satisfied**

- Touching one's own body
- Drawing one's own body
- Exercises with modelling clay (e.g. clay, modelling clay)
- Exercises with/in front of a mirror
- Exercises with video recordings of one's own body
- Exercises in everyday situations
- Exercises in which you imagine something (e.g. eating out)
- Other FREE TEXT

**Communication**

**How important do you think the following communication options with your therapist are? Please pick only one frequency per answer! Answer options: not important, less important, neutral, important, very important**

- Face-to-face
- By Phone
- By e-mail
- By video
- Other: FREE TEXT

**How important do you think the following communication options with other patients are? Please pick only one frequency per answer! Answer options: never, rarely, occasionally, often, very often**

- Face-to-face
- By Phone
- By e-mail
- By video
- Other: FREE TEXT

**How often are the following digital methods used in your Treatment? Please pick only one frequency per answer! Answer options: never, rarely, occasionally, often, very often**

- Virtual reality (e.g. 3D-Bodyscan)
- Wearables (small devices that are worn on the body, e.g. fitness bracelets, smart watches)
- Video calls (e.g. individual, group)
- Health apps
- E-Mail
- Telephone calls
- Social media, blogs (e.g. Facebook)
- Messenger apps (e.g. WhatsApp)
- Other FREE TEXT

**"Virtual reality" for the treatment of body image and body perception**

**The use of Virtual Reality (VR) creates a realistic environment in which patients and/or therapists can communicate with each other. This technique makes it possible to create an image of one's own body and to create different body silhouettes (e.g. normal weight). A virtual mirror then shows one's own body with corresponding body movements (see figures).**

**
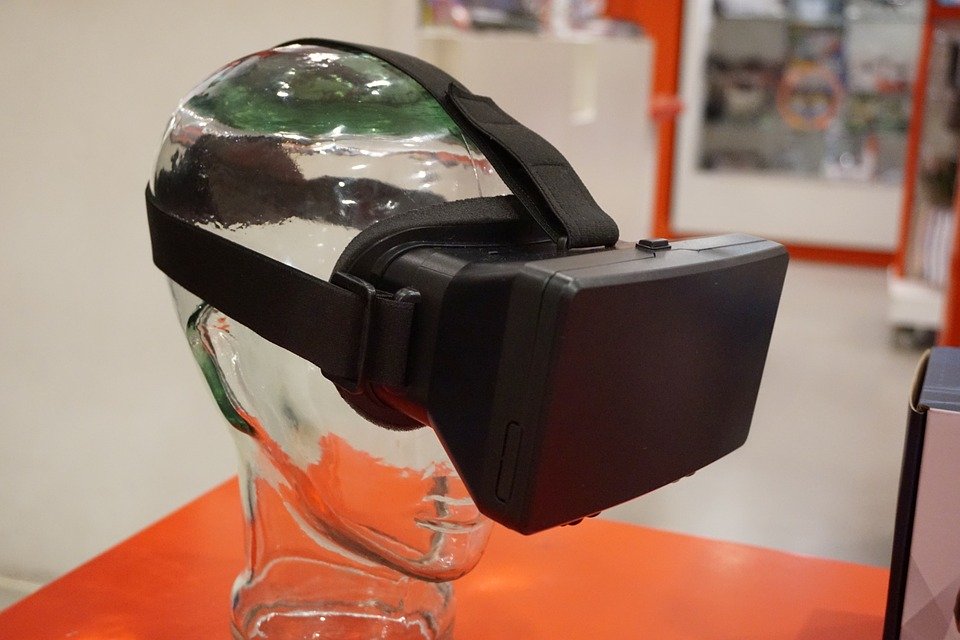
** © 2020 Pixabay

An example of VR glasses.

**
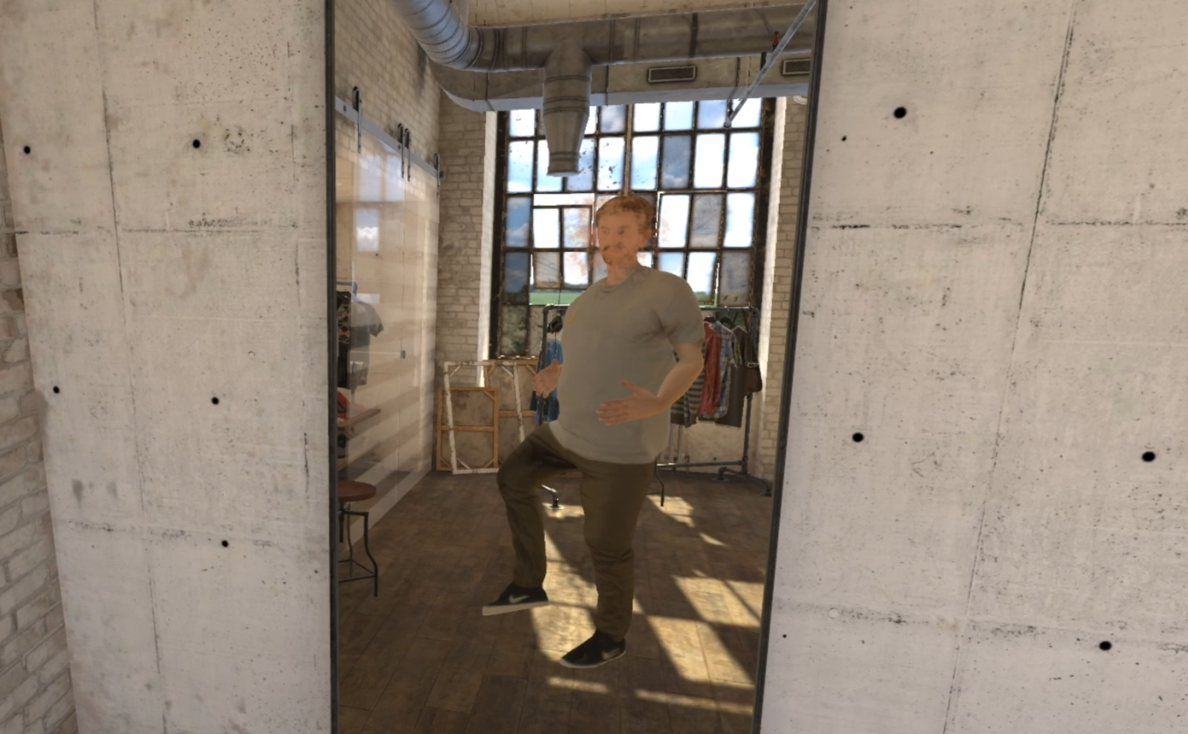
** © 2020 ViTraS

Exemplary representation through VR glasses. The patient can see his/her own body through a mirror.

**Have you already used VR-glasses as part of your current treatment?**

Yes

No

**Imagine your therapist suggesting VR glasses. To what extent would you agree with the following statements? Please pick only one frequency per answer!**

**Answer options: not applicable, rather not applicable, neutral, rather applicable, applicable**

- It would be easy for me to use VR glasses
- I would like to use VR glasses as part of the treatment

**Answer options: unsuitable, rather unsuitable, neutral, rather suitable, suitable.**

- I think the use of VR-glasses as part of an individual counselling session (=I'm alone with my therapist) is...?
- I think the use of VR-glasses as part of a group counselling session (=I'm with my therapist and several patients in a group) is...?

**Through VR, you have the opportunity to immerse yourself in different body shapes (e.g. normal weight, overweight). Realistic replicas of bodies and noticeable touching of one's own body enable a "real" perception of your body.**


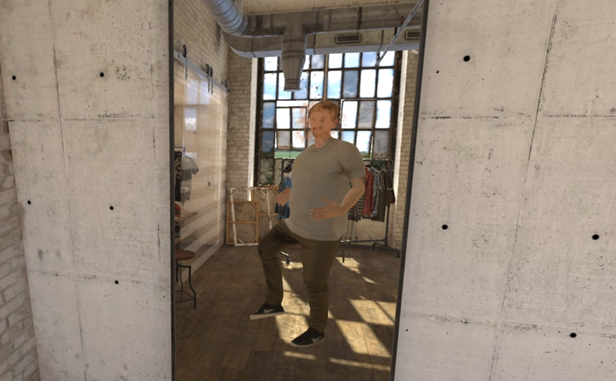
Example: Mr. B. is normal weight in reality. With VR glasses, he can recreate his body with excess weight.

**What do you think of the use of VR to support exercises intended to change body image perception?**

I think this is unsuitable

I think it is rather unsuitable

neutral

I think it is rather suitable

I think it is suitable

**In the context of virtual realities, it is possible for your therapist to "slip into" a virtual body. The therapist can then talk to you in real time (live), and you can see his/her facial expressions and gestures. Your therapist could also take on a different role for the counselling situation. What roles can you imagine for your therapist?” Answer options: agree/disagree**

- Friend
- Partner
- parents
- Children
- physician
- no role (just therapist)
- Other FREE TEXT

**Which potential advantages do you see in the use of VR as part of obesity treatment?**

FREE TEXT

**Which potential disadvantages do you see in the use of VR as part of obesity treatment?**

FREE TEXT

**How important is the inclusion of VR technology in the treatment of overweight/obesity in your opinion?**

Answer options: not important, less important, neutral, important, very important

**Sociodemographic information**

**What gender are you?**  *Please pick only* ***one*** *answer!*

|  | Female |  |  |
| --- | --- | --- | --- |
|  | Male |  |  |
|  | Other |  |  |

**How old are you?**

| Years |
| --- |

**Do you have German citizenship?** *Please pick only* ***one*** *answer!*

|  | Yes |
| --- | --- |
|  | No |

**In which state Life They?** *Please pick only* ***one*** *answer!*

|  | 16 federal states (dropdown menu) |
| --- | --- |

**What is your marital status?**  *Please pick only* ***one*** *answer!*

|  | Married and live with my spouse |
| --- | --- |
|  | Married and separated from my spouse |
|  | Single (e.g. single or partnership without marriage) |
|  | Divorced |
|  | Widowed |

**What is your highest general school leaving certificate?** *Please pick only* ***one*** *answer!*

|  | eighth grade or less |
| --- | --- |
|  | basic school leaving certificate |
|  | secondary school leaving certificate |
|  | high school diploma |
|  | college degree |

**Thank you for your participation!**
